# Immunohistopathological Analysis of Spongiosis Formation in Atopic Dermatitis Compared with Other Skin Diseases

**DOI:** 10.3390/dermatopathology12030023

**Published:** 2025-08-01

**Authors:** Ryoji Tanei, Yasuko Hasegawa

**Affiliations:** 1 Department of Dermatology, Tokyo Metropolitan Institute for Geriatrics and Gerontology, Itabashi, Tokyo 173-0015, Japan; 2 Department of Geriatric Pathology, Tokyo Metropolitan Institute for Geriatrics and Gerontology, Itabashi, Tokyo 173-0015, Japan; yhasegaw@tmig.or.jp

**Keywords:** allergic contact dermatitis, epidermal dendritic cell clusters, IgE-mediated atopic dermatitis, innate lymphoid cells, inflammatory dendritic epidermal cells, Langerhans cells, Pautrier collections, spongiosis, T cells, xerotic eczema

## Abstract

Whether the spongiotic reaction caused by the interaction of keratinocytes, T-lymphocytes, inflammatory dendritic epidermal cells (IDECs), and Langerhans cells (LCs) observed in atopic dermatitis (AD) represents a common feature of spongiosis in various skin diseases remains unclear. We analyzed the characteristics of spongiosis in AD compared with those in other eczematous dermatitis and inflammatory skin diseases by using immunohistochemical methods. Infiltration of IDECs (CD11c+ cells and/or CD206+ cells) and T-lymphocytes, accompanied by degenerated keratinocytes and aggregated LCs (CD207+ cells), was frequently observed as a common feature of spongiosis in multiple conditions. However, IDECs expressing IgE were identified exclusively in IgE-mediated AD. Aggregation of IDECs was predominantly observed in the spongiosis of adaptive immune-mediated eczematous disorders, such as AD and allergic contact dermatitis. These IDEC aggregations constituted the major components of the epidermal dendritic cell clusters seen in AD and other eczematous or eczematoid dermatoses, and may serve as a useful distinguishing marker from Pautrier collections seen in cutaneous T-cell lymphoma. These findings suggest that IDECs, in cooperation with other immune cells, may play a pivotal role in spongiosis formation in AD and various skin diseases, although the underlying immunopathological mechanisms differ among these conditions.

## 1. Introduction

Eczema and dermatitis, including allergic contact dermatitis (ACD), xerotic (asteatotic) eczema (XE), nummular eczema (NE), and atopic dermatitis (AD), have distinct etiologies and pathological mechanisms. However, they are collectively recognized as diseases that exhibit a common pathological reaction pattern of the skin, i.e., eczematous dermatitis. The histopathological hallmark of eczematous dermatitis is spongiotic dermatitis, which is characterized by intercellular edema with mononuclear cell infiltration in the epidermis, referred to as spongiosis, and inflammatory cell infiltration in the upper dermis, referred to as dermatitis [1]. Spongiosis in the epidermis is considered to result from keratinocyte apoptosis caused by the infiltrating mononuclear cells from the dermis and the associated impairment or loss of adhesion between keratinocytes, for example, through disruption of E-cadherin, allowing the influx of fluid (intercellular edema). In some cases, this process progresses to vesicle and bulla formation (spongiotic vesicles and bullae) [1,2]. Accordingly, spongiosis serves as a diagnostic marker in the histopathological evaluation of patients with eczema and dermatitis. Nevertheless, spongiosis is also observed in other inflammatory skin diseases, such as bullous pemphigoid (BP), where eosinophilic spongiosis is seen, and pigmented purpuric dermatosis (PPD) [3,4]. In addition, epidermal dendritic cell (DC) clusters, also referred to as pseudo-Pautrier microabscesses, Langerhans cell microgranulomas, Langerhans cell microvesicles, vase-shaped epidermal mononuclear cell collections, or epidermal DC aggregates, which represent a type of spongiosis, may be difficult to differentiate from Pautrier collections (also termed Pautrier microabscesses or Darier nests), which are characteristic of cutaneous T-cell lymphoma (CTCL) [1,3,5,6,7,8,9].

Recently, we demonstrated that in patients with immunoglobulin (Ig)E-mediated type AD, inflammatory dendritic epidermal cells (IDECs) and Langerhans cells (LCs), which express allergen-specific IgE, interact with keratinocytes and T-lymphocytes in an IgE-mediated delayed-type hypersensitivity reaction. This reaction may contribute to the formation of spongiotic dermatitis in AD lesions [1,10]. However, it remains unclear whether the spongiotic reaction involving the interaction of keratinocytes, T-lymphocytes, IDECs, and LCs is also present in the spongiosis of other eczematous dermatitis and other types of inflammatory skin diseases. Furthermore, it is unknown whether distinct differences can be identified in the spongiosis observed in AD compared with other eczematous and non-eczematous disorders, and whether epidermal DC clusters observed in eczematous dermatitis can be reliably differentiated from Pautrier collections observed in CTCL.

Therefore, in this study, we analyzed the characteristics of spongiosis formation in AD and various other skin diseases using immunohistochemical methods. The results showed that infiltration of IDECs and T-lymphocytes, accompanied by degenerated keratinocytes and aggregated LCs, was frequently observed in the spongiosis of multiple diseases. However, IgE-expressing IDECs were only detected in IgE-mediated AD. Aggregation of IDECs was prominently observed in the spongiosis of adaptive immune-mediated eczematous dermatitis, such as AD and ACD. These IDEC aggregations constituted the major component of epidermal DC clusters seen in eczematous dermatitis, and their presence may serve as a useful distinguishing feature from Pautrier collections seen in CTCL.

## 2. Materials and Methods

### 2.1. Subjects and Skin Samples

This study was conducted using skin biopsy specimens obtained from adult and older adult Japanese patients with various skin diseases who had been treated at our hospital from 1983 to 2024. The study protocols were approved by the Ethics Committee of the Tokyo Metropolitan Institute for Geriatrics and Gerontology (No. R15-42). The diagnosis of AD was established according to the clinical criteria of Hanifin and Rajka [11]. All skin biopsies were performed for diagnostic purposes, and samples from patients with AD were taken from active lichenified chronic lesions [1,10]. All patients, except for those from the 1980s to 2000s, provided written informed consent for each biopsy and for the use of their specimens for research. The use of biopsy specimens from earlier patients without written consent was approved by the hospital’s ethics committee.

### 2.2. Histological, Immunohistochemical, and Double-Immunofluorescence Staining

Formalin-fixed, paraffin-embedded, 3 μm thick sections were used for routine hematoxylin–eosin staining, single-immunohistochemical staining, and double-immunofluorescence staining. In some cases, frozen 7 μm thick sections were also prepared for single-immunohistochemical and double-immunofluorescence staining. Single-immunohistochemical staining was performed following the same procedures employed in our previous studies, and serial sections were prepared for analysis [10,12,13].

The following primary monoclonal antibodies (mAbs) and polyclonal antibodies (pAbs) were used: mouse mAbs against cluster of differentiation (CD)1a (LCs/IDECs, NCL-CD1a-235; Leica Biosystems Newcastle Ltd., Newcastle, U.K.), CD3 (T cells, LN10; Leica Biosystems, Tokyo, Japan), CD4 (helper/inducer/regulatory T cells, #713181; Nichirei Biosciences Inc., Tokyo, Japan), CD8 (cytotoxic T cells, #713201; Nichirei Biosciences Inc.), CD56 (natural killer (NK) cells, CD564; Leica Biosystems), CD68 (macrophages, N1576; Dako, Tokyo, Japan), CD206 (IDEC/macrophage, SC376232; Santa Cruz Biotechnology Inc., Santa Cruz, CA, USA), CD207 (LCs, ABIN1027332; antibodies-online Inc., Atlanta, GA, USA), mast cell (MC) tryptase (MCs, AA1; Abcam, Tokyo, Japan), 2D7 (basophils, ab155577; Abcam), IgE ε-chain (IgE, MH25-1; Santa Cruz Biotechnology Inc.), and interleukin (IL)-12 p70 (IL-12, SC74150; Santa Cruz Biotechnology Inc.); and rabbit mAbs against CD11c (dermal DCs/IDECs, EP1347Y; LSBio Inc., Seattle, WA, USA), IL-13 (IL-13, GTX37656; GeneTex Inc., Irvine, CA, USA); and rabbit pAbs against Der f1 (Mite Der f1; the main allergenic component of *Dermatophagoides farinae*; LB-7111; Cosmo Bio Ltd., Tokyo, Japan).

The dilution ratios for the primary antibodies were as follows: 1:30 for anti-CD1a, 1:50 for anti-IL-12 p70, 1:50 for anti-2D7, 1:100 for anti-CD206, and 1:200 for anti-IgE (mouse mAbs); 1:200 for anti-IL-13, and 1:250 for anti-CD11c (rabbit mAbs); and 1:1000 for anti-Der f1 (rabbit pAb). The other primary antibodies were used without dilution. Sections stained for IL-13 were developed with Vector Red (SK-5100; Vector Laboratories Inc., Newark, CA, USA), while the remaining sections were developed with 3,3′-diaminobenzidine (725191; Nichirei Biosciences Inc.) in the single-immunohistochemical staining.

Double-immunofluorescence staining was performed using paired mouse and rabbit mAbs for CD3 and IL-13. The procedure was based on methods previously described for frozen sections [10,12,13]. Briefly, after deparaffinization and hydrophilization of the paraffin sections, antigen retrieval was performed, followed by biotin blocking and blocking of non-specific binding. The sections were first incubated with the primary Abs for CD3 (mouse mAbs), followed by biotinylated anti-mouse IgG (BA-9200; Vector Laboratories Inc., Newark, CA, USA), and streptavidin–fluorescein conjugates (DyLight488 streptavidin, SA-5488; Vector Laboratories Inc.). Biotin blocking and blocking of non-specific binding were then repeated prior to incubation with the second primary Abs for IL-13 (rabbit mAbs), followed by biotinylated anti-rabbit IgG (BA-1000; Vector Laboratories Inc.) and streptavidin–fluorescein conjugates (DyLight594 streptavidin, SA-5594; Vector Laboratories Inc.). Nuclei were counterstained with 4′,6-diamidino-2-phenylindole (DAPI). The double-immunofluorescence-stained specimens were examined using a fluorescence microscope (BIOREVO BZ-9000/BZ-X810; Keyence, Osaka, Japan).

Combination double-immunofluorescence staining was performed using mouse mAbs for IgE and rabbit pAbs for Der f1, followed by immunohistochemical re-staining with anti-CD207 mAbs on a section obtained from a positive site of an atopy patch test (APT) for house dust mite (HDM) antigens (allergen extracts of *D. farinae;* Torii Pharmaceutical Co., Ltd., Tokyo, Japan) in one of the IgE-mediated AD cases. After image acquisition of the double-immunofluorescence staining on frozen sections, immunohistochemical re-staining was performed as follows: removal of the cover glass and thorough wash with distilled water; heat treatment (98 °C, 10 min) in 15 mM phosphate buffer (pH 6.0) to deactivate the primary and secondary antibodies from the double-immunofluorescence staining; followed by routine immunostaining procedures [13].

### 2.3. Evaluation of Immunohistopathological Findings

The analyses were conducted by qualitative and quantitative evaluation of the immunohistopathological findings on serial sections. In the quantitative evaluation, the number of immunopositive cells was counted under a microscope at 200× magnification. For spongiotic epidermis with focal spongiosis, immunopositive cells were counted within a 0.06 mm^2^ area. For epidermal DC clusters, immunopositive cells were counted within a 0.015 mm^2^ area. Immunopositive cells comprising Pautrier collections were similarly counted within multiple cellular nests, totaling 0.015 mm^2^. In this analysis, epidermal DC clusters were defined as dense cell clusters that were clearly separated from surrounding keratinocytes. The term epidermal DC aggregates was used comprehensively to refer to the pathological condition in which DCs (i.e., IDECs and LCs) gather within the spongiotic epidermis [1]. For each case, cell counts were obtained from the most cell-rich area among multiple examined fields. Only cells that were clearly positive for the immune-marker antigen of interest were counted. In this study, for the analysis of DCs in the epidermis, CD11c and CD206 were used as markers for IDECs, while CD207 was used as a marker of LCs [14,15]. CD1a, which is expressed on both IDECs and LCs, was treated as a common myeloid DC marker but is generally more strongly expressed in LCs [14]. For statistical analysis of the quantitative data, Welch’s *t*-test was used. Values of *p* < 0.05 were considered statistically significant. Data analysis was performed using EZR software (version 1.54; Saitama Medical Center, Jichi Medical University, Saitama, Japan).

## 3. Results

### 3.1. Clinical Data for Subjects and Preliminary Analysis

The clinical data for the subjects are summarized in Table 1. Patients receiving systemic immunosuppressive therapy or molecular targeted therapy were excluded. Topical treatments included either no treatment or topical corticosteroids; however, skin biopsies were performed in all cases due to a lack of clinical improvement. The analysis of patients with IgE-mediated AD was conducted on the same cases described in our previous study [10].

In a preliminary analysis using frozen sections with primary mAbs (2D7, ab 155577; Abcam plc.), which are not suitable for formalin-fixed sections, basophil infiltrate in the lichenified eczema of IgE-mediated AD was minimal and restricted to the dermis, with a low positive rate of 33.3% (2 of 6 cases). Therefore, basophils were excluded from further analyses of spongiosis formation in subsequent studies.

### 3.2. Immunohistopathological Findings of Spongiosis Formation in Atopic Dermatitis, Other Eczematous Dermatitis, and Other Types of Inflammatory Skin Diseases

#### 3.2.1. Atopic Dermatitis

- IgE-mediated atopic dermatitis (AD)

**Figure 1:**
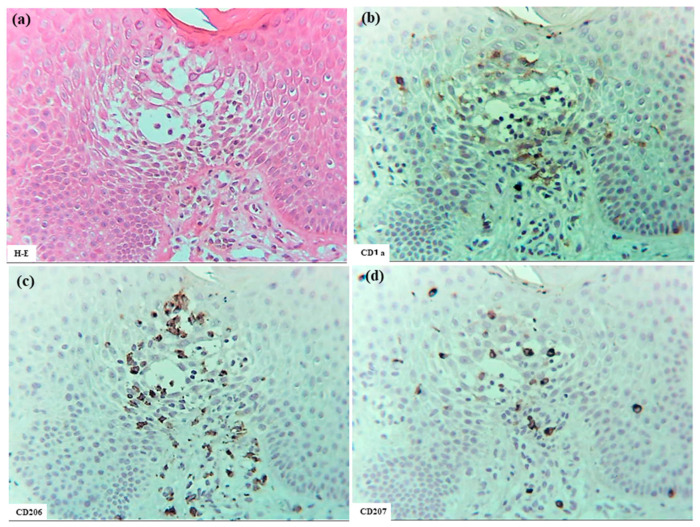

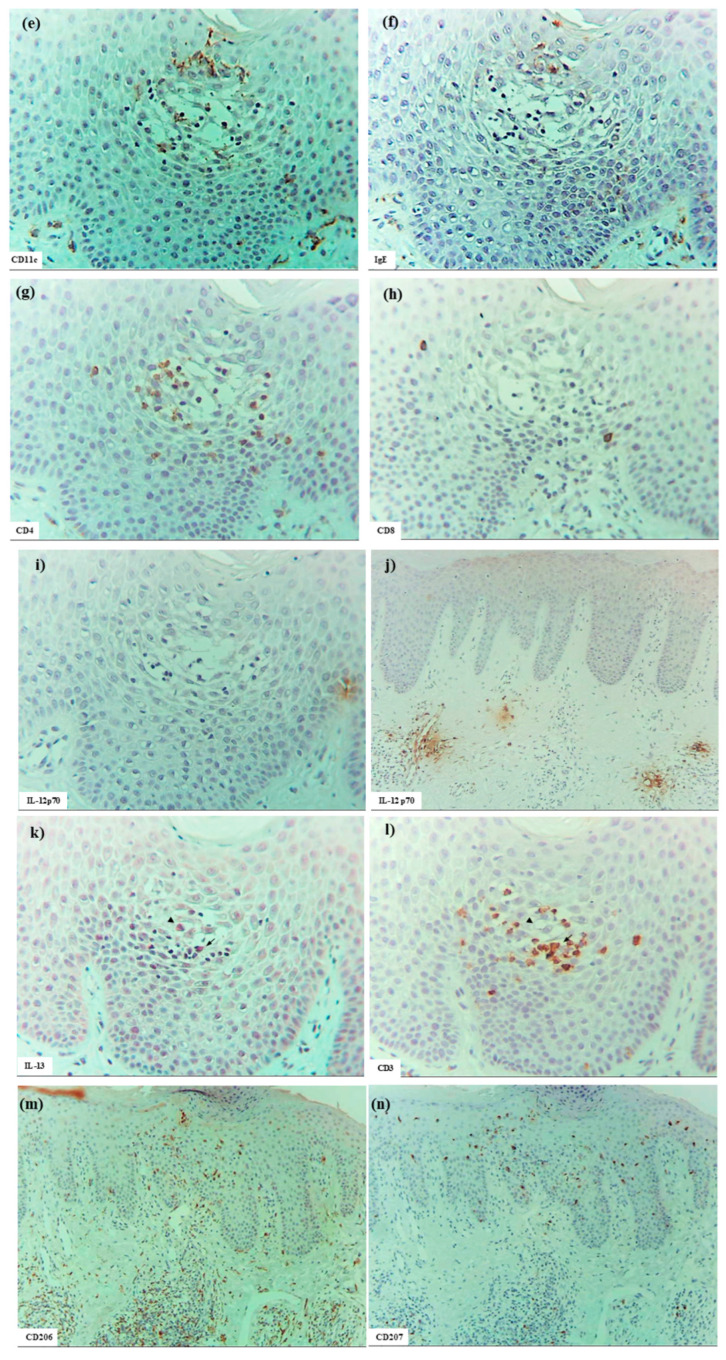

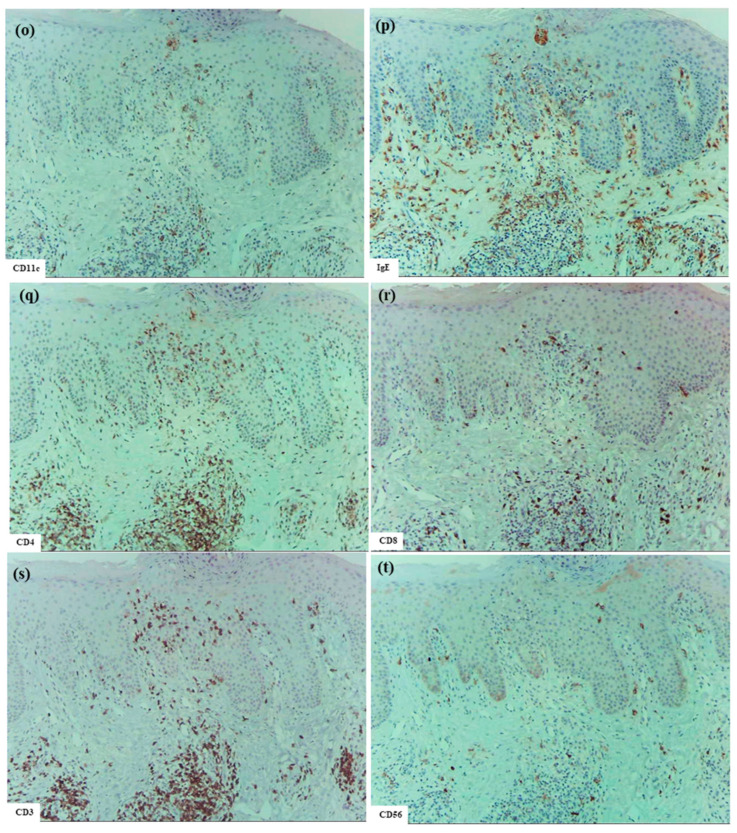
Spongiosis formation in IgE-mediated AD. (**a**) A spongiotic lesion showing intercellular edema, degenerated keratinocytes with elongation of intercellular bridges, and infiltration of mononuclear cells in the epidermis. (**b**) CD1a+ cells in the spongiotic epidermis. (**c**) CD206+ cells (i.e., IDECs) aggregating in the middle to upper layers of the spongiotic epidermis. (**d**) CD207+ cells (i.e., LCs) scattered in the spongiotic epidermis, frequently localized around IDECs. (**e**) CD11c+ cells, showing similar distribution to CD206+ cells, within the spongiotic epidermis. (**f**) IgE+ cells localized similarly to CD206+ and CD11c+ cells within the upper spongiotic epidermis. (**g**) Infiltrating CD4+ cells in the spongiotic epidermis. (**h**) CD8+ cells sparsely distributed at the periphery of the spongiotic epidermis. Note: In (**c**–**e**,**g**), some IDECs (CD206+ and CD11c+ cells) and LCs (CD207+ cells) are in contact with small mononuclear cells, while some infiltrating CD4+ cells are in contact with large mononuclear cells, suggesting potential interactions between these DCs and CD4+ lymphocytes within the spongiotic epidermis. (**i**) IL-12p70+ cells absent in the spongiotic epidermis. (**j**) IL-12p70+ cells localized focally around blood vessels in the upper dermis beneath the non-spongiotic epidermis. (**k**) IL-13+ cells in the spongiotic epidermis. (**l**) CD3+ cells in the spongiotic epidermis. Note: In (**k**,**l**), one IL-13+ cell (arrow) colocalizes with a CD3+ cell (arrow), while another IL-13+ cell (arrowhead) does not. (**m**–**t**) Representative images of CD206+ cells, CD207+ cells, CD11c+ cells, IgE+ cells, CD4+ cells, CD8+ cells, CD3+ cells, and CD56+ cells in the spongiotic epidermis and upper dermis, including iSALT-like structures. Note: In (**n**), CD207+ LCs are distributed widely in both spongiotic and non-spongiotic epidermis; in (**m**,**o**,**p**), IDECs (CD206+ cells, CD11c+ cells, and IgE+ cells) infiltrate and aggregate in the spongiotic epidermis. In (**q**–**t**), CD3+ CD4+ T cells predominate among infiltrating lymphocyte cells in both the spongiotic epidermis and the iSALT-like structures. (**a**): Case 2; hematoxylin–eosin staining. (**b**–**l**): Case 2; single-immunohistochemical staining. (**m**–**t**): Case 1; single-immunohistochemical staining. Panels (**a**–**i**,**k**–**t**) represent serial sections. Original magnifications: 100× (**j**,**m**–**t**); 200×, (**a**–**i**,**k**,**l**). Abbreviations: AD, atopic dermatitis; CD, cluster of differentiation; DC, dendritic cell; H-E, hematoxylin and eosin; IDEC, inflammatory dendritic epidermal cell; IgE, immunoglobulin E; IL, interleukin; iSALT, inducible skin-associated lymphoid tissue; and LC, Langerhans cell.

In six patients with IgE-mediated AD (Cases 1 to 6), hematoxylin–eosin and single-immunohistochemical staining were initially performed (Figure 1a–l for Case 2, Figure 1m–t: Case 1). The most prominent findings of spongiosis were the infiltration of CD3+ T cells together with CD206+ cells and CD11c+ cells, corresponding to IDECs, from the upper and papillary dermis to the acanthotic epidermis. Focal areas of intercellular edema and degenerated keratinocytes with elongation of intercellular bridges were also observed (Figure 1c,e,l,m,o,s). Parakeratotic scales were frequently present on the surface of the spongiotic epidermis. IDECs were located at the same sites as IgE+ cells within the epidermis (Figure 1f,p), although the number of IgE-expressing cells appeared slightly lower than previously reported using frozen sections [10]. IDECs (CD206+, CD11c+, and IgE+ cells) within the focal spongiosis tended to aggregate in the middle to upper layers of the spongiotic epidermis (Figure 1c,e,f,m,o,p). Conversely, CD207+ cells, corresponding to LCs, tended to gather around the IDECs (Figure 1c,d). Thus, CD207+ LCs were distributed widely throughout the spongiotic and non-spongiotic epidermis, with their numbers tending to increase within areas of spongiosis. In contrast, IDECs were predominantly seen within the spongiotic epidermis and only sparsely detected in the subepidermal layers of the non-spongiotic regions (Figure 1m–p). Analysis of serial sections revealed that, unlike frozen sections, IgE expression on LC was barely detectable in paraffin sections. CD1a+ cells were distributed in a pattern consistent with both LCs and IDECs (Figure 1b–f), although the expression intensity did not correlate strictly with IDEC localization [14]. CD4+ cells infiltrated mainly the middle to lower layers of the spongiotic epidermis, whereas CD8+ cells were scattered mainly at the periphery of the spongiotic areas (Figure 1g,h,q,r). Some IDECs and LCs were observed in close contact with small mononuclear cells, and CD4+ cells infiltrated in association with large mononuclear cells, suggesting interactions between DCs and CD4+T cells within the spongiotic epidermis (Figure 1c–e,g). Indeed, our previous study demonstrated that IgE-expressing IDECs coexisting with HDM antigens accumulated within the spongiotic epidermis of skin lesions in four (cases 1, 2, 5, and 6: 66.7%) of the six IgE-mediated AD patients sensitized to HDM, and these IDECs tended to localize in regions of CD4+ T-cell infiltration [10].

Interestingly, small numbers of CD56+ cells were also observed among the infiltrating cells in some areas of the spongiotic epidermis in all cases (Figure 1t) [16]. Serial selection analysis identified these CD56+ cells as CD56+ CD3- NK cells, a subset of group 1 innate lymphoid cells [1]. In contrast, CD68+ cells were primarily detected in the dermis. Only a small number of CD68+ cells were identified within the spongiotic epidermis in one case (Case 4), and serial section analysis suggested that most of these were CD68+ CD206- macrophages.

In cytokine analyses, IL-12p70 expression was not detected on the infiltrating cells or degenerating keratinocytes within the spongiotic epidermis. However, small focal areas of IL-12 p70+ keratinocytes were observed in the non-spongiotic epidermis in all cases [16]. In the upper dermis, IL-12p70+ cells were present focally near some blood vessels, but were infrequently located beneath the spongiotic epidermis (Figure 1i,j). Serial section analysis using immunostaining and hematoxylin–eosin indicated that the dermal IL-12p70+ cells were predominantly mononuclear cells, mostly monocytes or macrophages [16,17].

Meanwhile, infiltrating IL-13+ cells were observed in small numbers within the spongiotic epidermis (Figure 1k) in some cases, and in large numbers within the upper dermis in all cases. Serial section analysis indicated that the IL-13+ cells in the spongiotic epidermis included both CD3+ cells and CD3- cells (Figure 1k,l), while those in the upper dermis were comprised primarily of mononuclear cells, likely consisting of T cells, group 2 innate lymphoid cells (ILC2s) [18], and MCs [19].

**Figure 2:**
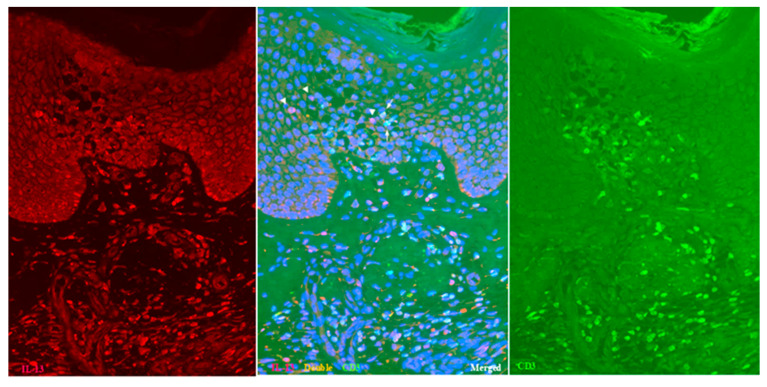
Spongiosis formation in IgE-mediated AD: double-immunofluorescent staining (Case 2). In addition to single-positive IL-13- CD3+ T cells (green), both double-positive IL-13+ CD3+ T cells (yellow: arrows) and single-positive IL-13+ CD3- cells (red: arrowheads) are observed among the infiltrating cells in the spongiotic epidermis. Double-positive IL-13+ CD3+ T cells (yellow) are also seen in the iSALT-like structures. Nuclei were labeled with DAPI (blue); single-channel DAPI images were omitted from the figures. Original magnification: 200×. Abbreviations: AD, atopic dermatitis; CD, cluster of differentiation; DAPI, 40,6-diamidino-2-phenylindole; IL, interleukin; iSALT, inducible skin-associated lymphoid tissue.

Double-immunofluorescent staining further confirmed the presence of IL-13+ CD3+ T cells and IL-13+ CD3- cells within the spongiotic epidermis (Figure 2; Case 2), in addition to IL-13- CD3+ T cells. As immunostaining for MC tryptase showed that MCs were essentially confined to the dermis [12], the IL-13+ CD3- cells observed in the spongiotic epidermis were likely ILC2s [1,18]. In the upper dermis, numerous IL-13+ CD3+ T cells and IL-13+ CD3- cells also infiltrated beneath the spongiotic epidermis (Figure 2).

In the upper dermis beneath the spongiotic epidermis, clusters of inflammatory infiltrating cells corresponding to inducible skin-associated lymphoid tissues (iSALTs) [20] were frequently observed. Recent studies have highlighted the importance of iSALTs (a term originally used to describe the responses in ACD) and iSALT-like structures (a provisional term used for analogous structures in other inflammatory skin disorders) in the establishment of effector phases of adaptive cutaneous immune responses [20,21,22]. Hematoxylin–eosin and single-immunohistochemical staining (Figure 1m–t) demonstrated that the clusters in IgE-mediated AD were composed mainly of T cells (CD3+, CD4+, and CD8+ cells), DCs (CD11c+, IgE+, CD206+, and CD207+ cells), and macrophages (CD68+ and CD206+ cells). Among T cells, CD4+ cells were considerably more prevalent than CD8+ cells. Double-immunofluorescence analysis further indicated that IL-13+ CD3+ T cells constituted a subset of those CD4+ (CD3+) cells within the clusters (Figure 2) [1]. In addition, some CD56+ NK cells were present within the clusters. MCs (MC tryptase+ cells), which were generally widely distributed throughout the papillary and upper dermis, were frequently localized at the periphery of the clusters. Occasionally, a few eosinophils were also observed. IgE+ cells, consisting primarily of MCs and DCs, were found in large numbers in the cellular infiltrates of the dermis, including iSALT-like structures. Based on the morphological distribution of IgE+ IDECs previously demonstrated by double-immunofluorescence staining [1,10], it may be speculated that many of the IDECs (CD206+, CD11c+, and IgE+ cells) identified in the spongiotic epidermis had infiltrated from these iSALT-like structures (Figure 1m,o,p).

Additionally, in two cases (Cases 2 and 6), uninvolved skin from patients with IgE-mediated AD was examined. In these samples, LCs (CD207+ cells) were present in a regular array within the normal-thickness epidermis, lacking spongiosis, whereas IDECs (CD206+ and/or CD11c+ cells) were absent or detected only sporadically at the basal layer.

- Atopy patch test in a patient with IgE-mediated atopic dermatitis

**Figure 3:**
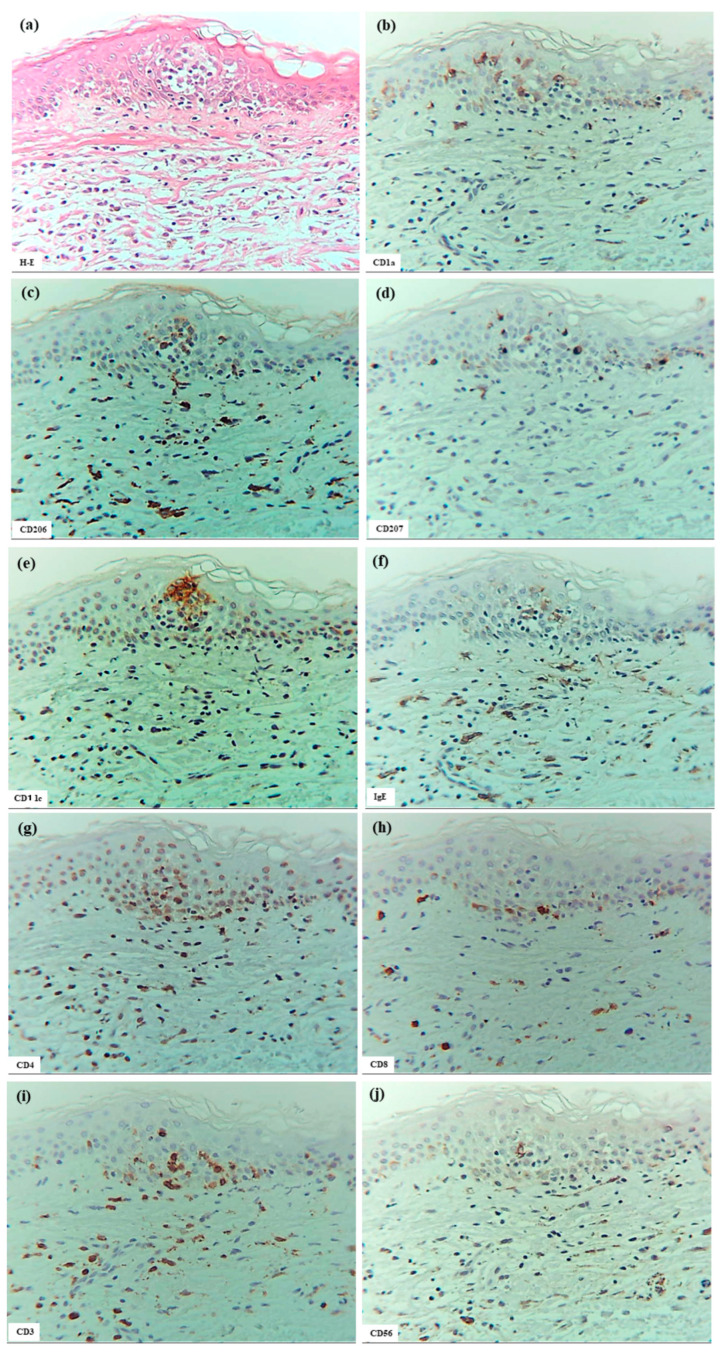

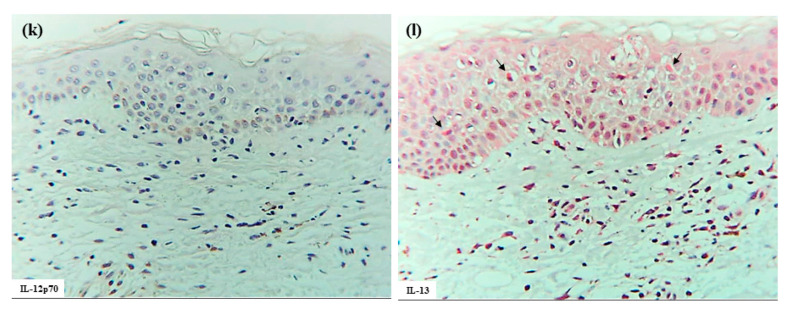
Spongiosis formation at a positive-reaction site of APT against HDM antigens (*D. farinae*) in a patient with IgE-mediated AD. (**a**) Spongiosis in eczematous erythema at the positive APT site. (**b**–**j**) CD1a+ cells, CD206+ cells, CD207+ cells, CD11c+ cells, IgE+ cells, CD4+ cells, CD8+ cells, CD3+ cells, and CD56+ cells in the spongiotic epidermis. Note: In (**c**–**j**), CD206+ and CD11c+ cells (i.e., IDECs) aggregate in the middle to upper layers of the spongiotic epidermis; CD207+ cells (i.e., LCs) are primarily localized around the IDECs; the number of IgE+ cells is lower than that of CD206+ and CD11c+ cells; CD4+ and CD3+ cells infiltrate the spongiotic epidermis; and CD56+ cells (i.e., NK cells) are also present in the spongiotic epidermis. (**k**) IL-12p70+ cells are not detected in the spongiotic epidermis. (**l**) A few IL-13+ cells with red-stained cytoplasm (arrows) are observed in another area of spongiotic epidermis. (**a**–**l**): Case 5; H-E and single-immunohistochemical staining. Panels (**a**–**k**) represent serial sections. Original magnifications: 200×. Abbreviations: AD, atopic dermatitis; APT, atopy patch test; CD, cluster of differentiation; D, Dermatophagoides; H-E, hematoxylin and eosin; IDEC, inflammatory dendritic epidermal cell; IgE, immunoglobulin E; IL, interleukin; LC, Langerhans cell; and NK, natural killer.

Analyses were performed on a patient (Case 5) who exhibited a positive reaction to an APT against HDM antigens (allergen extracts of *D. farinae*). Spongiosis formation in the eczematous erythema at the positive APT site was observed (Figure 3a–l), showing features comparable to those seen in the lichenified eczema of IgE-mediated AD. Specifically, focal spongiosis was seen in the acanthotic epidermis; CD3+ CD4+ T cells and IDECs (CD206+ cells and/or CD11c+ cells) infiltrated the spongiotic epidermis; IDECs tended to aggregate in the middle to upper layers of the spongiotic epidermis; CD207+ LCs were primarily localized around the aggregating IDECs; and IgE+ cells were present at the same sites as IDECs (CD206+ and/or CD11c+ cells), though their numbers were low. Additionally, small numbers of CD56+ CD3- NK cells and IL-13+ cells were observed in some areas of the spongiotic epidermis. IL-12p70+ cells were not detected in the spongiotic epidermis, although small focal areas of IL-12 p70+ keratinocytes were present in non-spongiotic epidermis. CD68+ cells were observed only in the dermis. A notable difference from lichenified eczema of IgE-mediated AD was the milder degree of epidermal acanthosis and absence of prominent iSALT-like structures in the upper dermis beneath the spongiotic epidermis.

Double-immunofluorescence staining indicated the presence of IL-13- CD3+ T cells, IL-13+ CD3+ T cells, and IL-13+ CD3- cells (presumably ILC2s) in the spongiotic epidermis.

**Figure 4:**
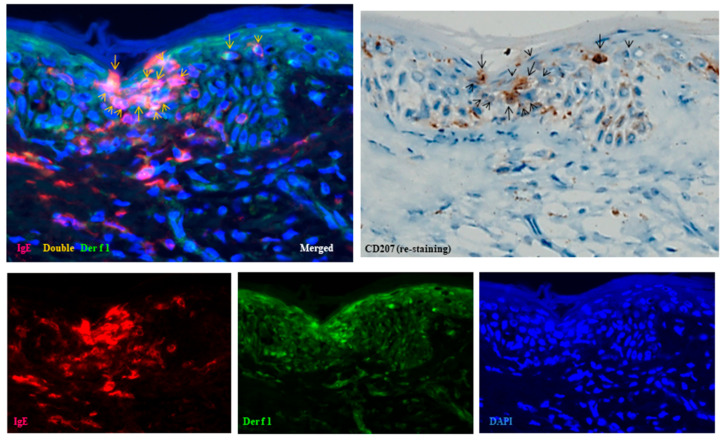
Spongiosis formation at a positive-reaction site of APT against HDM antigens (*D. farinae*) in a patient with IgE-mediated AD: combination of double-immunofluorescence staining and immunohistochemical re-staining (Case 5). Double-immunofluorescence staining shows focal spongiosis with accumulation of double-positive IgE+ cells and Der f1+ cells (yellow images indicated by yellow arrows and arrowheads). Immunohistochemical re-staining demonstrates that both IgE+ CD207+ cells (LCs: black arrows) and IgE+ CD207- DCs (IDECs: black arrowheads) have captured HDM antigens (Der f1). Original magnification: 200×. These results have been previously reported in a review article [1] and are republished here with permission from the authors and publisher. Abbreviations: AD, atopic dermatitis; APT, atopy patch test; CD, cluster of differentiation; D, Dermatophagoides; DAPI, 40,6-diamidino-2-phenylindole; DC, dendritic cell; IDEC, inflammatory dendritic epidermal cell; IgE, immunoglobulin E; and LC, Langerhans cell.

Furthermore, combined double-immunofluorescence staining and immunohistochemical re-staining demonstrated that, in the early focal spongiosis of eczematous erythema caused at the positive APT site, IDECs and LCs expressing IgE and capturing HDM antigens (Der f1) accumulated within the spongiotic epidermis [1]. Initial double-immunofluorescence staining identified focal spongiosis with accumulations of double-positive IgE+ cells and Der f1+ cells. Subsequent immunohistochemical re-staining demonstrated that both IgE+ CD207+ cells (LCs) and IgE+ CD207- DCs had captured HDM antigens (Figure 4). Since IgE+ DCs in the epidermis are typically either LCs or IDECs [10], the IgE+ CD207- DCs were considered to represent IgE+ IDECs.

Those findings suggest that the IDECs and LCs, after capturing HDM antigens, presented them to infiltrating CD4+ T cells, interacted with other lymphoid cells, and thereby contributed to the progression or resolution of spongiosis formation.

- Non-IgE-mediated atopic dermatitis

Analysis was performed on one case (Case 7). The spongiosis formation observed in non-IgE-mediated AD exhibited features generally similar to those in IgE-mediated AD. However, several differences were noted. No IgE+ cells and only a few IL-13+ cells were detected in the spongiotic epidermis. In contrast, infiltration of CD8+ T cells and CD56+ NK cells was more prominent compared to IgE-mediated AD.

#### 3.2.2. Xerotic Eczema

**Figure 5:**
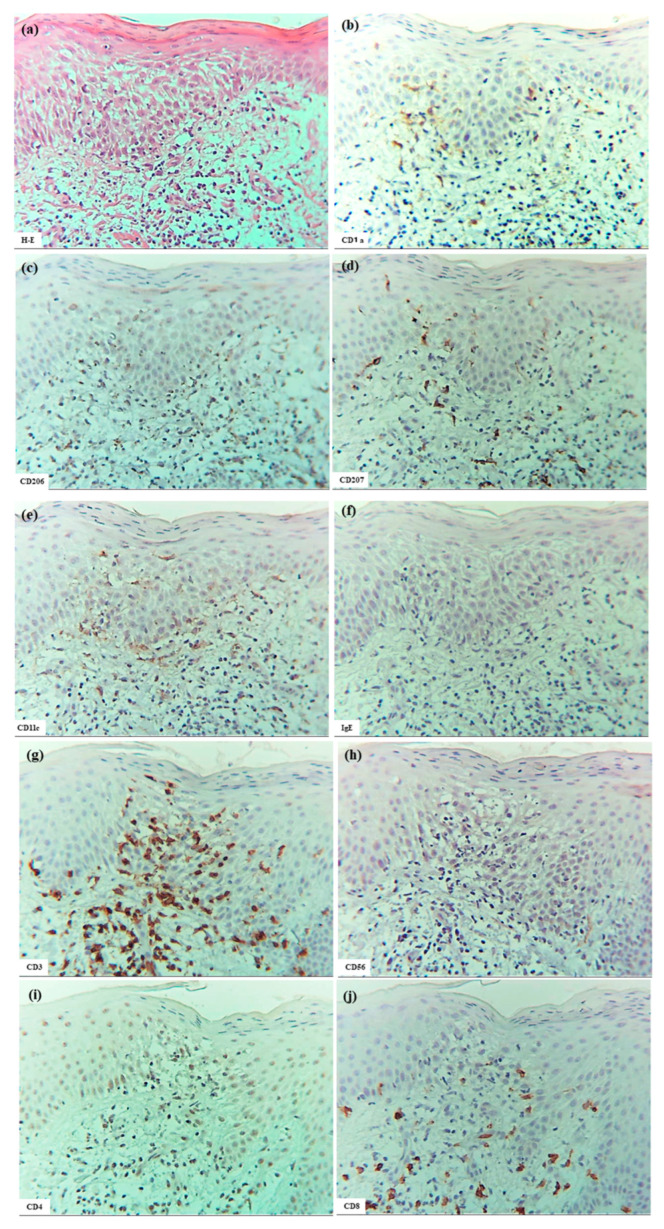
Spongiosis formation in XE. (**a**–**j**) Focal spongiosis observed in XE. Note: in (**a**–**f**), some CD207+ LCs and CD11c+ IDECs are present in the spongiotic epidermis; however, the expression of CD206 on IDECs is weak. Note: In (**g**–**j**), infiltration of CD4+ cells is less prominent compared to CD3+ cells within the spongiotic epidermis. Several CD8+ cells and a few CD56+ cells (NK cells) are present within the focal spongiosis. (**a**–**j**): Case 8; H-E and single-immunohistochemical staining. Panels (**a**–**j**) represent serial sections. Original magnification: 200×. Abbreviations: CD, cluster of differentiation; H-E, hematoxylin and eosin; IDEC, inflammatory dendritic epidermal cell; LC, Langerhans cell; NK, natural killer cell; and XE, xerotic eczema.

In XE (Cases 8 to 13, with the latter three cases partially exhibiting nummular-form eczema), which developed from senile xerosis (SX), spongiosis formation generally resembled that observed in IgE-mediated AD (Figure 5). Specifically, CD3+ T cells and IDECs (CD206+ and/or CD11c+ cells) infiltrated the focal spongiosis of the acanthotic epidermis. Occasional CD56+ NK cells were present within the spongiotic epidermis; IL-12p70+ cells were not detected in the spongiotic epidermis, however, in some cases, IL-12p70+ cells were present focally near blood vessels in the upper dermis. Infiltration of IL-13+ cells was also evident in the upper dermis in some cases. CD68+ cells were primarily located in the dermis. Several notable differences from IgE-mediated AD were observed. Acanthosis was milder than in AD; spongiotic vesicles and superficial crusting of the epidermis occurred more frequently; infiltration of CD4+ cells and IDECs (CD206+ and/or CD11c+ cells) was less prominent compared to those of CD8+ cells and CD207+ LCs in the spongiotic epidermis; IDECs rarely formed aggregates, and epidermal DC clusters were not observed; few IL-13+ cells were observed in the spongiotic epidermis; and IgE-expressing cells were restricted to a subset of MCs in the dermis. Regarding CD207+ LCs, infiltration into focal spongiotic areas varied, ranging from marked accumulation to decreased infiltration compared to non-spongiotic epidermis. Focusing on infiltrating lymphocytes revealed that the relative numbers of CD4+, CD8+, and CD3+ cells suggested a possible preferential infiltration of CD3+ CD4- CD8- double-negative T cells [23] in the spongiotic epidermis of XE.

Additionally, in two cases (Cases 8 and 12), SX, which is the underlying cause of XE, was analyzed. No spongiosis was observed in the epidermis, and cellular infiltration in the dermis was minimal. In the epidermis of SX, LCs (CD207+ cells) were arranged in regular arrays, similar to normal skin, while IDECs (CD206+ and/or CD11c+ cells) were absent or detected in very small numbers.

#### 3.2.3. Allergic Contact Dermatitis and Systemic-Type Contact Dermatitis

**Figure 6:**
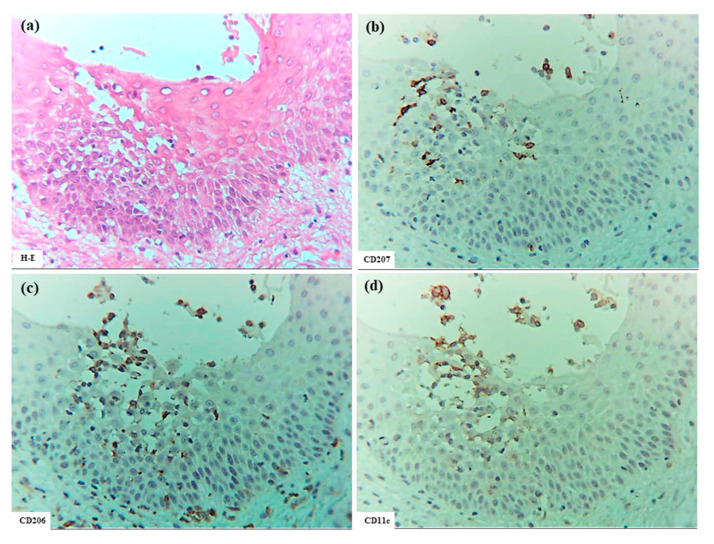

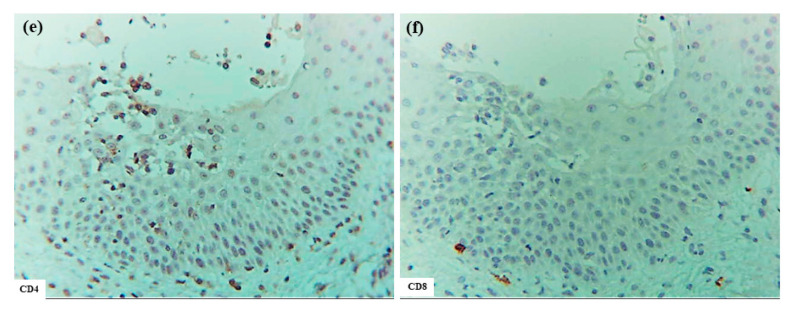
Spongiosis formation in ACD. (**a**) Spongiotic epidermis located beneath a spongiotic bulla. (**b**–**f**) CD207+ cells, CD206+ cells, CD11c+ cells, CD4+ cells, and CD8+ cells in the spongiotic epidermis. Note: In (**b**–**f**), IDECs (CD206+ and/or CD11c+ cells) infiltrate and aggregate in the area from the upper spongiotic epidermis to the lower bulla; CD207+ LCs are localized mainly around the IDECs; CD4+ T cells tend to infiltrate the same areas where IDECs and LCs are present; and few CD8+ T cells are observed in the spongiotic epidermis. (**a**–**f**): Case 14; H-E and single-immunohistochemical staining. Panels (**a**–**f**) represent serial sections. Original magnification: 200×. Abbreviations: ACD, allergic contact dermatitis; CD, cluster of differentiation; H-E, hematoxylin and eosin; IDEC, inflammatory dendritic epidermal cell; and LC, Langerhans cell.

In ACD (Cases 14 to 18), spongiosis formation was observed over a broader area than in IgE-mediated AD, and was occasionally accompanied by eosinophil infiltration into the epidermis. The inflammatory response was prominent not only in the epidermis but also extended into the hair follicle epithelium, with marked formation of spongiotic vesicles and bullae, particularly in cases caused by plants (Figure 6a–f). Similar to AD, infiltration of T cells (CD3+, CD4+, and CD8+ cells), IDECs (CD206+ and/or CD11c+ cells), and LCs (CD207+ cells) into the spongiotic epidermis was consistently observed, although the predominant cell populations varied between cases. IDECs lacked IgE expression in all cases. CD56+ NK cells and IL-13+ cells were also observed within the spongiotic epidermis in some cases. In a few cases, small numbers of CD68+ cells were present in the spongiotic epidermis, but these cells showed a different infiltration pattern from CD206+ cells. In the cases caused by plant allergens, IDECs, LCs, CD4+ cells, and CD3+ cells infiltrated the spongiotic vesicles and bullae. iSALT-like structures were consistently observed in the upper dermis beneath the spongiotic dermis.

As in IgE-mediated AD, expression of IL-12p70 was not detected in most areas of the spongiotic epidermis in ACD. However, in 3 out of 5 cases, IL-12p70 expression was observed in the peripheral regions of some spongiotic epidermis, occasionally located directly beneath epidermal DC clusters. IL-12p70 expression was observed in small areas containing degenerated keratinocytes, with or without infiltrating IDECs, LCs, and CD4+ cells within the spongiotic epidermis. However, precise identification of IL-12p70-producing cells was not performed in the present analysis. Additionally, small areas of IL-12p70 expression were observed in non-spongiotic epidermis and within perivascular infiltrates in the upper dermis in most cases.

In SCD, a generalized form of ACD, spongiosis formation similar to that observed in ACD was also present.

#### 3.2.4. Other Forms of Eczematous Dermatitis

- Nummular eczema (NE)

Analysis was performed on three cases (Cases 20 to 22) of NE with a dispersed form, unrelated to XE or AD. Compared to IgE-mediated AD, spongiosis formation in NE showed a more intense inflammatory response, with marked infiltration of T cells (CD3+, CD4+, and CD8+ cells) and IDECs (CD206+ and/or CD11c+ cells) across a broader area of acanthotic epidermis with spongiotic changes. The distribution of LCs (CD207+ cells) varied among cases, showing either increased or decreased infiltration. IDECs did not express IgE; however, some formed aggregates in the upper layers of the spongiotic epidermis, extending towards the stratum corneum. In one case (Case 20), infiltration of CD68+ cells, displaying a distinct infiltration pattern from CD206+ cells, was observed, and infiltration of CD56+ NK cells was also prominent. A small number of IL-13+ cells were detected within the spongiotic epidermis, while IL-12p70 expression was not observed in any case.

#### 3.2.5. Other Types of Inflammatory Skin Diseases

- Pigmented purpuric dermatosis

In two cases (Cases 23 and 24) of PPD with focal spongiosis, the features of spongiosis formation were generally similar to those observed in IgE-mediated AD and other eczematous disorders. The characteristic findings were as follows: focal spongiosis with infiltration of CD3+ T cells and IDECs (CD206+ and/or CD11c+ cells) was mainly observed in the lower to middle layers of the epidermis; CD207+ LCs were present in these areas; both CD4+ and CD8+ cells infiltrated the epidermis prominently; marked infiltration of CD4+, CD8+, and CD3+ cells was observed in the upper dermis; and small iSALT-like structures were frequently present in the upper dermis beneath the spongiotic epidermis. Expression of IL-12p70 was not detected; however, a few IL-13+ cells were observed in one case (Case 24), and CD56+ NK cells were identified within the spongiotic epidermis in both cases. An interesting finding was the presence of an epidermal DC cluster beneath the stratum corneum in one case (Case 24), as described in detail below. Notable differences from lichenified eczema in IgE-mediated AD included the milder degree of epidermal acanthosis, absence of IgE expression in IDECs, and abundant extravasation of red blood cells both around the iSALT-like structures and within the infiltrates in the spongiotic epidermis.

- Bullous pemphigoid

Analysis was performed on one case (Case 25), in which eosinophilic spongiosis was observed in the skin lesions of BP. The notable findings included multiple large and small spongiotic vesicles with abundant eosinophil infiltration within the epidermis. Prominent infiltration of IDECs (CD206+ and/or CD11c+ cells), CD3+, and CD4+ cells was also observed in the spongiotic epidermis surrounding the vesicles; however, IDECs did not exhibit aggregation within the epidermis. Scattered LCs (CD207+ cells) and CD8+ cells were also identified in the spongiotic epidermis. Infiltration of CD68+, CD56+, and IL-13+ cells was minimal, and expression of IL-12p70 was not detected.

### 3.3. Immunohistopathological Findings of Epidermal Dendritic Cell Clusters in Eczematous Dermatitis Versus Pautrier Collections in Cutaneous T-Cell Lymphoma

#### 3.3.1. Epidermal Dendritic Cell Clusters

- IgE-mediated atopic dermatitis

**Figure 7:**
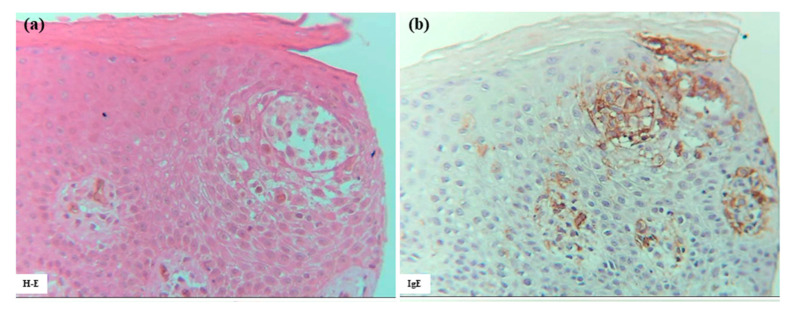

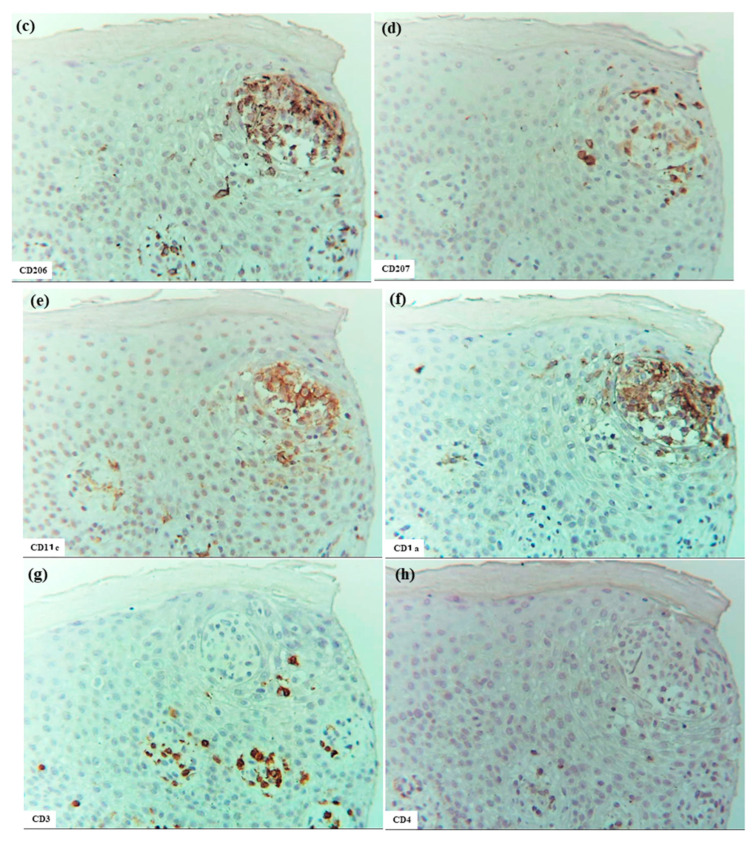
Epidermal DC clusters in IgE-mediated AD. (**a**–**h**) An epidermal DC cluster located in the upper middle layer of the spongiotic epidermis on the right side of the section. Note: In (**b**–**h**), the cluster is mainly composed of CD206+ CD11c+ IgE+ IDECs, with a small number of CD207+ LCs. Constituent cells of the cluster show strong CD1a expression. CD3+ and CD4+ cells are absent from the cluster. (**a**–**h**): Case 3; H-E and single-immunohistochemical staining. Panels (**a**–**h**) represent serial sections. Original magnification: 200×. Abbreviations: AD, atopic dermatitis; CD, cluster of differentiation; DC, dendritic cell; H-E, hematoxylin and eosin; IDEC, inflammatory dendritic epidermal cell; IgE, immunoglobulin E; and LC, Langerhans cell.

Several large and small epidermal DC clusters were observed in one case (Case 3). As noted in previous reports [3,5,23], the epidermal DC clusters in IgE-mediated AD lesions were typically located from the upper middle layer to just beneath the stratum corneum, in and around the spongiotic epidermis. These clusters were primarily composed of CD206+ CD11c+ IDECs, with a smaller population of CD207+ LCs. These IDECs tended to express CD1a and IgE strongly. Infiltration of CD3+, CD4+, CD8+, CD56+, and IL-13+ cells into the clusters was absent or minimal, and IL-12p70 + cells were not detected (Figure 7).

- Allergic contact dermatitis and systemic-type contact dermatitis

**Figure 8:**
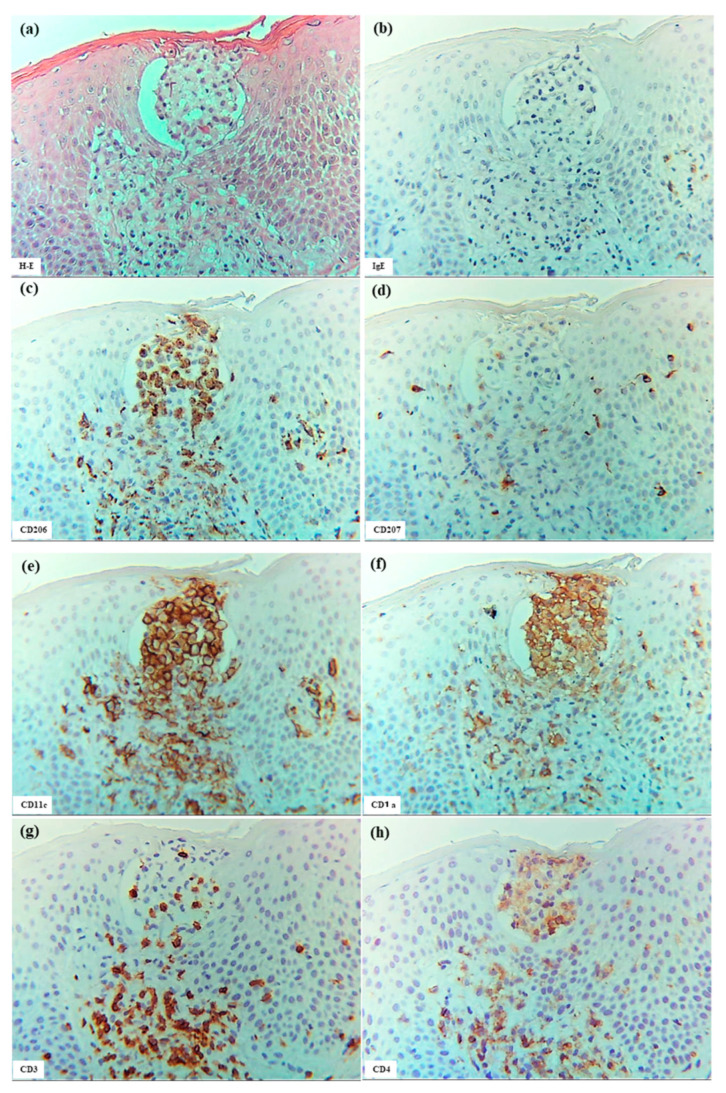

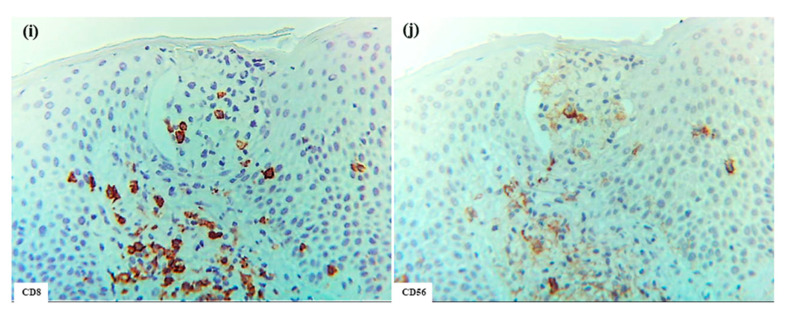
Epidermal DC clusters in SCD. (**a**) An epidermal DC cluster observed within the spongiotic epidermis. (**b**–**j**) Immunohistochemical findings for IgE+, CD206+, CD207+, CD11c+, CD1a+, CD3+, CD4+, CD8+, and CD56+ cells in the cluster. Note: In (**a**–**j**), the cluster is primarily composed of CD206+ CD11c+ CD1a+ IDECs. Several CD4+ and CD3+ cells, and a few CD8+, CD56+, and CD207+ cells were also observed in the cluster. The cluster is located just beneath the stratum corneum and is predicted to eventually be expelled from the epidermis [13], having formed wide spaces between itself and surrounding keratinocytes. Note: In (**c**,**e**,**f**,**h**), IDECs (CD206+ CD11c+ CD1a+ cells) within the cluster show faint expression of CD4 antigen. (**a**–**h**): Case 19; H-E and single-immunohistochemical staining. Panels (**a**–**j**) represent serial sections. Original magnification: 200×. Abbreviations: CD, cluster of differentiation; DC, dendritic cell; H-E, hematoxylin and eosin; IDEC, inflammatory dendritic epidermal cell; IgE, immunoglobulin E; LC, Langerhans cell; and SCD, systemic-type contact dermatitis.

In ACD (Cases 15 and 18) and SCD (Case 19), epidermal DC clusters were observed with features similar to those seen in IgE-mediated AD. These clusters were mainly composed of CD206+ CD11c+ IDECs, with a small number of CD207+ LCs. CD1a expression was clearly observed in two cases, but was barely observed in one case (Case 15). Several notable differences from the clusters observed in IgE-mediated AD were identified. Epidermal DC clusters in ACD and SCD were occasionally accompanied by infiltration of CD3+, CD4+, CD8+, CD56+ NK cells, and eosinophils. Clusters were present not only in the epidermis but also within the hair follicle epithelium. IgE expression was absent from IDECs in all cases. A small number of IL-13+ cells were found within the clusters in two cases (Cases 18 and 19). Interestingly, IDECs comprising the epidermal DC clusters in ACD and SCD occasionally expressed CD4 antigen faintly [24], although at a much lower intensity than that observed on CD4+ T cells. IL-12p70+ cells were not observed within the epidermal DC cluster in any case; however, as noted above, IL-12p70 expression was observed in the spongiotic epidermis directly beneath an epidermal DC cluster in one case (Case 18). The differences in the characteristics of epidermal DC clusters between IgE-mediated AD and ACD/SCD may reflect differences in the pathophysiology and/or differences in the stage of cluster formation at the time of observation (Figure 8).

- Pigmented purpuric dermatosis

In one case of PPD (Case 24), an epidermal DC cluster similar to those observed in ACD and SCD was identified. The epidermal DC cluster, composed primarily of CD206+ CD11c+ CD1a+ IDECs with a few CD207+ CD1a+ LCs, was located just beneath the stratum corneum within a spongiotic area of the epidermis. The IDECs in the cluster also faintly expressed CD4-antigen and were accompanied by CD4+, CD8+, CD3+, and CD56+ cells within the cluster. IL-13+ cells and IL-12p70 expression were not evident within the cluster. These findings, which resemble those seen in ACD/SCD, may suggest that cell-mediated immune responses contribute to the pathogenesis of PPD and indicate a possible association with ACD [4].

#### 3.3.2. Pautrier Collections in Cutaneous T-Cell Lymphoma

**Figure 9:**
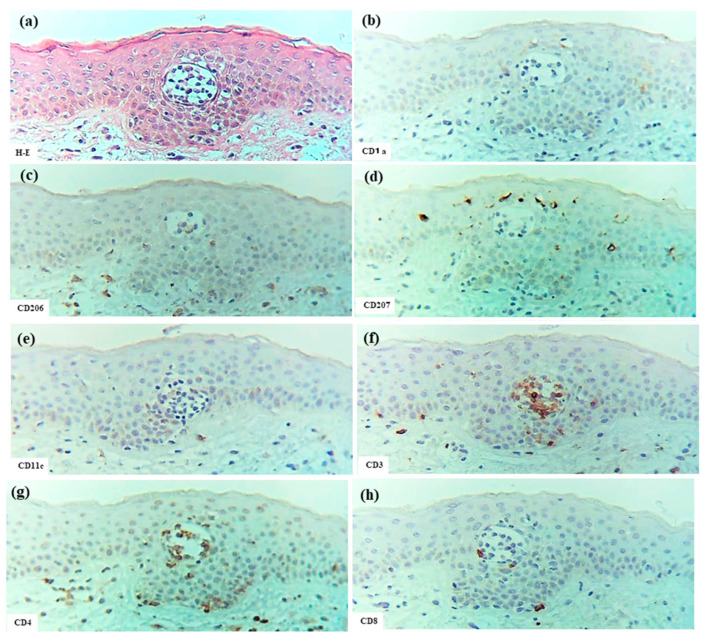
Pautrier collections in CTCL associated with ATL. (**a**) Pautrier collection consisting of a nest of atypical mononuclear cells located in the central part of the epidermis. (**b**–**h**) Immunohistochemical findings for CD1a+, CD206+, CD207+, CD11c+, CD3+, CD4+, and CD8+ cells within the nest. Note: In (**a**–**h**), the nest is composed primarily of CD4+ CD3+ T cells. (**a**–**h**): Case 30; H-E and single-immunohistochemical staining. Panels (**a**–**h**) represent serial sections. Original magnification: 200×. Abbreviations: ATL, adult T-cell leukemia/lymphoma; CD, cluster of differentiation; CTCL, cutaneous T-cell lymphoma; and H-E, hematoxylin and eosin.

In the present analysis of CTCL, all cases represented adult T-cell leukemia/lymphoma (ATL) with cutaneous involvement [25]. The characteristics of Pautrier collections were consistent across cases and included the following features: multiple nests of atypical mononuclear cells composed of CD3+ cells were scattered throughout the epidermis; most cells within the nests were CD4+ cells, but CD8+ cells were observed in small numbers; a few CD207+ cells were detected within some nests; only occasional CD206+, CD11c+, and CD1a+ cells were observed; and CD56+ cells and IgE+ cells were not detected within these collections (Figure 9)

### 3.4. Quantitative Evaluations of Immunohistopathological Findings

#### 3.4.1. Spongiotic Formation in IgE-Mediated Atopic Dermatitis, Xerotic Eczema, and Allergic Contact Dermatitis

Statistical analyses of the spongiotic dermatitis were performed in cases of IgE-mediated AD, XE, and ACD, which have relatively well-defined pathological etiologies (Table 2). In comparisons of cell numbers within the spongiotic epidermis, IgE+ cells, i.e., representing approximately IgE-expressing IDECs, were significantly more numerous in IgE-mediated AD than in XE and ACD. CD206+ and CD4+ cells were significantly more numerous in IgE-mediated AD than in XE. When comparing IgE-mediated AD with ACD, infiltration of CD4+, CD8+, and CD3+ cells into the spongiotic epidermis tended to be greater in ACD, although these differences did not reach statistical significance.

The immunohistopathological characteristics of each disease were as follows. In IgE-mediated AD, although no statistically significant difference was observed, IDECs (CD11c+ and/or CD206+ cells) were more abundant than LCs (CD207+ cells) in the spongiotic epidermis across all cases. Furthermore, CD4+ cells were significantly more numerous than CD8+ cells. In XE, LCs (CD207+ cells) tended to be more prevalent than IDECs (CD11c+ and/or CD206+ cells); CD4+ cells tended to be less abundant than CD8+ cells; and CD4+ cells were significantly less abundant than CD3+ cells in the spongiotic epidermis. In ACD, although differences among individual cases were observed, there was an overall tendency for IDECs (CD11c+ and/or CD206+ cells) to be more prevalent than LCs (CD207+ cells) in the spongiotic epidermis. In cases of IgE-mediated AD, XE, and ACD, IL-13- CD3+ cells were significantly more numerous than IL-13+ CD3+ cells or IL-13+ CD3- cells.

#### 3.4.2. Epidermal Dendritic Cells Clusters in Eczematous Dermatitis Versus Pautrier Collections in Cutaneous T-Cell Lymphoma

**Figure 10:**
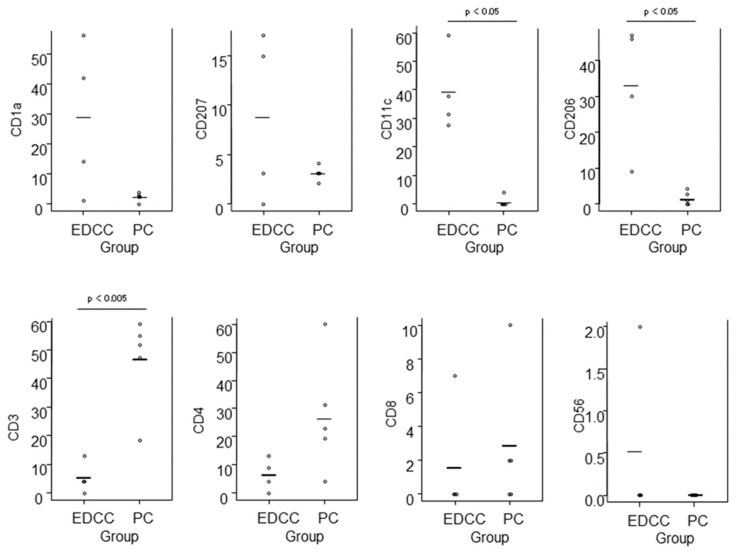
Comparison of immunohistochemically stained cell counts between epidermal DC clusters in eczematous dermatitis and Pautrier collections in CTCL. Cell counts were performed in 0.015 mm^2^ areas of epidermal DC clusters and in aggregated areas totaling 0.015 mm^2^ within Pautrier collections. Horizontal bars indicate mean values. Abbreviations: CD, cluster of differentiation; CTCL, cutaneous T-cell lymphoma; DC, dendritic cells; EDCC, epidermal dendritic cell clusters; and PC, Pautrier collections.

Epidermal DC clusters and Pautrier collections were compared between the four cases of eczematous dermatitis (IgE-mediated AD: 1 case, ACD: 2 cases, and SCD: 1 case) and five cases of ATL. For the analysis of epidermal DC clusters, we selected clusters with higher cell density and larger size, and counted only CD4+ cells with clearly positive expression. In summary (Figure 10), quantitative comparison revealed that epidermal DC clusters contained significantly greater numbers of CD11c+ and CD206+ cells, i.e., IDECs, while Pautrier collections contained significantly greater numbers of CD3+ cells, i.e., T-lymphocytes. There was also a tendency for epidermal DC clusters to contain more CD1a+ cells compared to Pautrier collections; however, this difference did not reach statistical significance, as CD1a expression was barely detectable in one case of ACD.

## 4. Discussion

In previous studies, we demonstrated that IDECs and LCs expressing allergen-specific IgEs mediate an IgE-dependent delayed-type hypersensitivity reaction in response to environmental allergens (e.g., HDMs), and that this reaction can be observed in spongiotic dermatitis within lichenified eczema of adults and older adults with IgE-mediated AD [1,10,13]. Similar to ACD [20,21,22,26], delayed-type hypersensitivity reactions to antigens and allergens appear to contribute to the pathophysiology of IgE-mediated AD. However, allergen-specific IgE may exert additional “booster”-type effects, such as heightened sensitivity and amplification of the hypersensitivity response, potentially contributing to the chronicity and severity of AD [1,10,27,28,29]. Thus, delayed-type hypersensitivity may represent a common underlying mechanism in the spongiotic dermatitis of allergic eczematous disorders.

In the present study, we analyzed the features of spongiosis formation in AD, particularly IgE-mediated AD, in comparison with other forms of eczematous dermatitis and other types of inflammatory skin diseases. In addition, we examined the characteristic differences between the epidermal DC clusters seen in eczematous dermatitis and Pautrier collections observed in CTCL. The results indicated that infiltration of IDECs (CD206+ and/or CD11c+ cells) and T-lymphocytes (CD3+ cells), accompanied by aggregation of LCs (CD207+ cells) and degenerated keratinocytes within the acanthotic epidermis exhibiting intercellular edema, was a commonly observed feature of spongiosis across these diseases. However, reductions in LC numbers within the spongiotic epidermis were noted in certain disorders (e.g., XE and ACD). Aggregated IDECs were mainly found in the adaptive immune eczematous disorders (e.g., AD and ACD/SCD), while IgE-expressing IDECs were found exclusively in IgE-mediated AD. Furthermore, the results suggest that the IDEC aggregations represent the major cellular component of epidermal DC clusters seen in eczematous dermatitis and that their presence may serve as a useful distinguishing feature to differentiate these clusters from Pautrier collections in CTCL. However, it is important to note that the presence of epidermal DC clusters itself is not a definitive marker of benign disease, as spongiosis accompanied by epidermal DC clusters is occasionally observed in CTCL [6].

One of the identification markers for IDECs, i.e., CD206, is a C-type lectin receptor that recognizes mannose-type glycans and is involved in phagocytosis of foreign substances and antigen presentation [14]. A recent study by Manome-Zenke et al. [30] suggested that IDECs infiltrating the spongiotic epidermis in AD lesions also express another receptor, macrophage galactose-type C-type lectin (MGL). In contrast, LCs do not express CD206 or MGL [14,30]; however, their identifying marker, CD207/Langerin, is also a C-type lectin receptor [31]. In our previous analysis, as in APT-induced AD-like lesions, it was demonstrated that in lichenified eczema, IgE-expressing IDECs that had captured HDM antigens infiltrated and accumulated in the spongiosis epidermis together with CD4+ cells in approximately 60% of patients with IgE-mediated AD and HDM allergy (same patients in the present study) [1,10]. The analysis also indicated that the co-expression of HDM antigens and IDECs diminished as aggregation progressed and spongiotic vesicles developed, a process that coincided with reduced infiltration of CD4+ cells at that stage [1,13]. These findings may suggest that, as components of the adaptive immune response in the skin, IDECs, which represent a type of monocyte-derived inflammatory DC, recognize glycan structures present on HDMs and other antigens via C-type lectin receptors [32], internalize these antigens through phagocytosis them, just as macrophages do, and then present them to CD4+ T-lymphocytes within the spongiotic epidermis, ultimately contributing to antigen clearance [1]. In contrast, in the present analysis of XE, CD206 expression on IDECs was weaker, CD4+ T-cell infiltration was less prominent in the spongiotic epidermis, IDEC aggregation was infrequent, and epidermal DC clusters were not observed. These findings may reflect that phagocytosis of foreign substances and antigen presentation by IDECs are not substantially involved in the pathophysiology of XE, a condition in which the adaptive immune system appears to play a limited role [33]. Furthermore, as previous study have suggested a possible role for cellular immunity in cases of XE progressing to NE form [34], infiltration of CD3+ CD4- CD8- (double-negative) T cells, which have been reported to be involved in both innate and adaptive immunity [23], may contribute to modulating immune responses involved in spongiosis formation in the present XE cases.

In general, interferon (INF)-γ-secreting CD4+ T helper (Th)1 cells are considered to play a key role in the formation of spongiosis in eczematous dermatitis, with IL-12 being required for their activation. However, the skin manifestations of AD are characterized by the predominance of type-2 cytokine (IL-4/IL-13, etc.) immune responses, driven mainly by ILC2 cells and CD4+ Th2 cells. Macrophages, eosinophils, dermal DCs, and IDECs have all been reported as major sources of IL-12 production in AD lesions [1,16,17,35,36]. Interestingly, in the six cases of IgE-mediated AD with HDM allergy analyzed in the present study, no IL-12p70 expression was detected in IDECs within the spongiosis epidermis or in IDECs forming epidermal DC clusters, despite previous confirmation that these IDECs had captured HDM antigens in four of the six cases (cases 1, 2, 5, and 6) [1,10]. Furthermore, in the present study, no IL-12p70 expression was observed on IDECs in the spongiotic epidermis at sites positive for HDM in APT-induced lesions, even though these IDECs had captured HDM antigens. These findings contrasted with previous in vitro and ex vivo studies, which reported the production and secretion of IL-12p70 by IDECs [36]. This discrepancy may reflect differences in analytical methodologies, but it may also suggest that in IgE-mediated AD, IDECs infiltrating the spongiotic epidermis may no longer express significant levels of IL-12p70, which induces Th1 responses, despite capturing, internalizing, and presenting antigens to T-lymphocytes within these lesions.

As reported in a previous study [18], our analysis of lichenified eczema and eczematous erythema at positive APT sites in IgE-mediated AD demonstrated that, in addition to IL-13- CD3+ (CD4+) T cells (Th1 cells), small numbers of IL-13+ CD3+ (CD4+) T cells (i.e., Th2 cells) and IL-13+ CD3- lymphoid cells (presumably ILC2s) infiltrated some areas of the spongiotic epidermis. Furthermore, a few CD56+ NK cells were also identified among the infiltrating cells in some of the spongiotic epidermis. These findings suggest that both Th1 and Th2 cells may be involved in spongiosis formation in AD as components of adaptive immunity [1], and that NK cells and ILC2s might also participate as components of innate immunity [18].

Although not examined in the present analysis, conflicting findings have been reported regarding the involvement of CD4+ CD25+ Forkhead box protein P3 (Foxp3)+ regulatory T (Treg) cells in spongiosis formation. Szegedi et al. [5] reported the presence of CD4+ CD25+ Foxp3+ Treg cells within epidermal DC clusters at APT-positive sites and within spongiotic lesions of AD. In contrast, Verhagen et al. [37] reported that CD4+ CD25+ Foxp3+ Treg cells were absent from AD lesions and APT-positive sites, but observed considerable infiltration of IL-10-secreting CD4+ Treg type-1 cells (Tr1 cells), which can suppress allergen-specific activation of both INF-γ-secreting Th1 cells and IL-4-secreting Th2 cells. In our analysis, CD3+ CD4+ cells were rarely detected within the epidermal DC clusters in IgE-mediated AD, suggesting that neither Foxp3+ Treg cells nor Tr1 cells were major constituent cells within the mature stage of epidermal DC clusters, particularly in clusters that do not yet exhibit prominent detachment from surrounding keratinocytes.

In the present study, we confirmed that spongiosis formation, characterized by infiltration of IDECs, T-lymphocytes, and LCs, together with degenerated keratinocytes and intercellular edema, as observed in IgE-mediated AD, also occurs in other allergic and non-allergic skin diseases. These findings suggest that spongiosis formation may develop not only through mechanisms involving delayed-type hypersensitivity reactions but also through interactions among other immune and biological processes [38]. Additionally, it has been noted that in the pathophysiology of allergic skin diseases like ACD, the molecular profiles of delayed-type hypersensitivity reactions may vary depending on the specific causative allergen or antigen involved [39]. Further studies are needed on these aspects in the future.

This study has several limitations. These include inherent technical limitations associated with immunohistopathological analyses, the potential for downregulation or loss of cell markers and cytokines in formalin-fixed paraffin-embedded sections, the absence of blinding in quantitative evaluations, and the relatively small sample sizes for AD and the other disease groups. Although the present study focused on diseases with relatively well-defined etiologies, spongiosis formation also occurs in a variety of skin disorders with complex or uncertain causes, which were not examined in this study. In addition, this study was conducted as a morphological observational analysis and did not account for time-dependent changes in spongiosis formation across the disease course. While we believe that biopsies captured spongiosis formation at a representative stage of disease activity, it is likely that histopathological characteristics will vary depending on the severity of inflammation and the timing of skin sampling.

## 5. Conclusions

The findings of the present study suggest that IDECs, in collaboration with keratinocytes, T-lymphocytes, LCs, and other immune cells, may play a pivotal role in spongiosis formation in AD and in various other skin diseases, despite clear differences in their respective immunopathologies. Furthermore, IDEC aggregations constitute the primary cellular component of the epidermal DC clusters observed in the spongiotic dermatitis of skin lesions in AD and other forms of eczematous dermatitis. The presence of these IDEC aggregations may serve as a useful distinguishing marker for differentiating epidermal DC clusters in eczematous dermatitis from Pautrier collections in CTCL.

## Figures and Tables

**Table 1:** Clinical data of the subjects.

Case	Age/Sex	Disease	Biopsy Site	Remarks	Cont. Case	Cont. Age/Sex	Cont. Disease	Cont. Biopsy Site	Cont. Remarks
1	78/M	IgE-mediated AD	Forearm	Allergic to HDMs, etc.	14	80/F	ACD	Thigh	Allergen: plants
2	61/F	IgE-mediated AD	Forearm	Allergic to HDMs, etc. APT-positive ^†^	15	91/F	ACD	Face	Allergen: plants
3	83/F	IgE-mediated AD	Thigh	Allergic to HDMs, etc.	16	82/F	ACD	Face	Allergen: cosmetics
4	49/M	IgE-mediated AD	Abdomen	Allergic to HDMs, etc.	17	77/F	ACD	Face	Allergen: eyeglass
5	84/M	IgE-mediated AD	Upper back	Allergic to HDMs, etc. APT-positive ^†^	18	52/M	ACD	Sole	Allergen: shoes
6	40/M	IgE-mediated AD	Chest	Allergic to HDMs, etc.	19	69/M	SCD	Forearm	Allergen: dialysis-related medicines
7	81/F	Non-IgE- mediated AD	Forearm	Serum total and specific IgEs: WNL	20	73/M	NE	Lower leg	Dispersed form
					21	70/M	NE	Upper back	Dispersed form
					22	34/F	NE	Lower leg	Dispersed form
8	78/M	XE	Lower leg	Caused by SX	23	71/F	PPD	Lower leg	With venous stasis
					24	81/F	PPD	Lower leg	Unknown cause
9	83/M	XE	Lower leg	Caused by SX	25	77/M	BP	Sole	ES Serum anti-BP 180NC16a Ab: positive
10	72/M	XE	Side chest	Caused by SX	26	72/F	CTCL	Trunk	Disease type: ATL
					27	68/F	CTCL	Lower leg	Disease type: ATL
11	75/M	XE	Lower leg	Caused by SX, partially NE-form	28	66/F	CTCL	Lower back	Disease type: ATL
					29	46/F	CTCL	Face	Disease type: ATL
					30	47/M	CTCL	Neck	Disease type: ATL
12	76/M	XE	Forearm	Caused by SX, partially NE-form					
13	54/M	XE	Upper arm	Caused by SX, partially NE-form					

^†^ APT-positive for HDM antigens. Abbreviations: Ab, antibodies; ACD, allergic contact dermatitis; AD, atopic dermatitis; APT, atopy patch test; ATL, adult T-cell leukemia/lymphoma; BP, bullous pemphigoid; CTCL, cutaneous T-cell lymphoma; ES, eosinophilic spongiosis; F, female; HDM, house dust mite; IgE, immunoglobulin E; M, male; NE, nummular eczema; PPD, pigmented purpuric dermatosis; SCD, systemic-type contact dermatitis; SX, senile xerosis; XE, xerotic eczema; and WNL, within normal limits.

**Table 2:** Immunohistochemical staining results for the quantitative analyses of infiltrating cells in spongiotic epidermis of patients with IgE-mediated atopic dermatitis, xerotic eczema, and allergic contact dermatitis.

Case and Disease				Single-IHC Staining						Double-IF Staining			
Infiltrating Cells in Spongiotic Epidermis ^§^													
	IgE+ Cells	CD11c+ Cells	CD206+ Cells	CD207+ Cells	CD4+ Cells	CD8+ Cells	CD3+ Cells	CD56+ Cells	IL-12p70+ Cells	IL-13+ Cells	IL-13-/CD3+ Cells	IL-13+/CD3+ Cells	IL-13+/CD3- Cells
1 IgE-AD	45	31	47	23	47	19	54	5	0	0	56	0	0
2 IgE-AD	10	13	45	15	25	2	33	4	0	2	16	2	3
3 IgE-AD	20	38	37	9	16	6	14	6	0	1	16	1	1
4 IgE-AD	18	20	21	9	36	14	37	0	0	0	21	4	1
5 IgE-AD	9	12	12	10	30	1	17	0	0	0	14	3	0
6 IgE-AD	16	12	8	7	17	15	15	5	0	2	12	7	2
Mean	19.7	21	28.3	12.2	28.5	9.5	28.3	3.3	0	0.8	22.5	2.8	1.2
(±SD)	(±13.2)	(±11.1)	(±16.9)	(±5.9)	(±11.8)	(±7.5)	(±15.9)	(±2.6)	(±0)	(±0.9)	(±16.7)	(±2.5)	(±1.1)
	*, **		*		*								
8 XE	0	11	5	15	6	11	26	3	0	0	54	0	0
9 XE	0	10	10	37	1	7	38	2	0	2	48	4	0
10 XE	0	2	6	5	0	30	24	7	0	0	25	0	0
11 XE	0	26	13	57	11	6	87	6	0	0	64	0	0
12 XE	0	14	3	35	0	11	25	0	0	0	24	0	0
13 XE	0	11	16	19	3	4	14	0	0	0	18	0	1
Mean	0	12.3	8.8	28.0	3.5	11.5	35.7	3.0	0	0.3	38.8	0.7	0.2
(±SD)	(±0)	(±7.8)	(±5.0)	(±18.7)	(±4.3)	(±9.5)	(±26.3)	(±3.0)	(±0)	(±0.8)	(±18.9)	(±1.6)	(±0.4)
14 ACD	0	36	54	21	30	1	24	1	0	2	35	2	0
15 ACD	0	52	32	20	50	10	48	2	0	8	35	18	6
16 ACD	0	16	50	7	55	62	74	24	0	0	64	0	0
17 ACD	0	0	13	21	0	22	21	9	0	0	29	0	0
18 ACD	0	47	13	24	90	54	131	16	4	2	48	4	3
Mean	0	30.2	32.4	18.6	45.0	29.8	59.6	10.4	0.8	2.4	42.2	4.8	1.8
(±SD)	(±0)	(±21.8)	(±19.5)	(±6.7)	(±33.1)	(±26.9)	(±45.2)	(±9.7)	(±1.7)	(±3.2)	(±14.0)	(±7.5)	(±2.6)

The immune markers indicate the following cell populations within the spongiotic epidermis: IgE+ cells are almost all IDECs, with only a small portion being LCs; CD11c+ and CD206+ cells are IDECs; CD207+ cells are LCs; CD4+ cells are primarily helper T cells; CD8+ cells are primarily cytotoxic T cells; CD3+ cells are mature T cells; CD56+ cells are primarily NK cells; IL-12p70+ and IL-13+ cells express the respective cytokines; IL-13-/CD3+ cells are T cells that do not express IL-13; IL-13+/CD3+ cells are IL-13-expressing T cells; and IL-13+/CD3- cells are IL-13-expressing non-T cells (presumably ILC2s). ^§^ Cell counts were performed in the most cell-rich area of a 0.06 mm^2^ region of spongiotic epidermis. Data are presented as the mean ± standard deviation. * *p* < 0.05 compared to XE. ** *p* < 0.05 compared to ACD. Abbreviations: ACD, allergic contact dermatitis; AD, atopic dermatitis; CD, cluster of differentiation; IDECs, inflammatory dendritic epidermal cells; IF, immunofluorescence; IgE, immunoglobulin E; IgE-AD, IgE-mediated AD; IHC, immunohistochemical; IL, interleukin; ILC2s, group 2 innate lymphoid cells; LCs, Langerhans cells; NK, natural killer; and XE, xerotic eczema.

## Data Availability

Data is contained within the article. The original contributions presented in this study are included in the article. Further inquiries can be directed to the corresponding author.
